# Taxonomic Resolutions Based on 18S rRNA Genes: A Case Study of Subclass Copepoda

**DOI:** 10.1371/journal.pone.0131498

**Published:** 2015-06-24

**Authors:** Shu Wu, Jie Xiong, Yuhe Yu

**Affiliations:** 1 Key Laboratory of Aquatic Biodiversity and Conservation of Chinese Academy of Sciences, Institute of Hydrobiology, Chinese Academy of Sciences, Wuhan, China; 2 University of Chinese Academy of Sciences, Beijing, China; Consiglio Nazionale delle Ricerche (CNR), ITALY

## Abstract

Biodiversity studies are commonly conducted using 18S rRNA genes. In this study, we compared the inter-species divergence of variable regions (V1–9) within the copepod 18S rRNA gene, and tested their taxonomic resolutions at different taxonomic levels. Our results indicate that the 18S rRNA gene is a good molecular marker for the study of copepod biodiversity, and our conclusions are as follows: 1) 18S rRNA genes are highly conserved intra-species (intra-species similarities are close to 100%); and could aid in species-level analyses, but with some limitations; 2) nearly-whole-length sequences and some partial regions (around V2, V4, and V9) of the 18S rRNA gene can be used to discriminate between samples at both the family and order levels (with a success rate of about 80%); 3) compared with other regions, V9 has a higher resolution at the genus level (with an identification success rate of about 80%); and 4) V7 is most divergent in length, and would be a good candidate marker for the phylogenetic study of *Acartia* species. This study also evaluated the correlation between similarity thresholds and the accuracy of using nuclear 18S rRNA genes for the classification of organisms in the subclass Copepoda. We suggest that sample identification accuracy should be considered when a molecular sequence divergence threshold is used for taxonomic identification, and that the lowest similarity threshold should be determined based on a pre-designated level of acceptable accuracy.

## Introduction

In recent years, there have been significant advances in the rationale, methodology, and application of molecular taxonomy [1–4]. Of these methods, DNA barcoding is utilized for species identification and discovery, using a short DNA marker [5,6]. It has been demonstrated that the COI gene is a suitable DNA barcode for animal species identification, though it may not be suitable for the resolution of classification at higher taxonomic levels [7–9]. Identification at higher taxonomic levels would be needed in at least the following three cases: (1) barcoding database is incomplete, (2) inter-specific distance is insufficient for species identification, and (3) newly discovered taxa need to be classified. Moreover, the use of DNA barcoding may be limited by insufficient sampling [3], or the overlap of intra- and inter-specific variability; thus, species identifications based only on DNA barcoding of a single-locus marker remain inaccurate [10–14]. Combining mitochondrial genes with nucleic genes would produce more credible species delineations than those based on a single gene [14,15]. One of the candidate nuclear markers is the small nuclear subunit of the ribosomal RNA (SSU rRNA) gene, which is a common molecular marker frequently used in phylogenetic studies [16–19]. In eukaryotes, the nuclear 18S rRNA gene is also popularly used in diversity research [20–27]. Compared to COI, evolution progresses much more slowly in the 18S rRNA gene, potentially making it a more valuable markers for distinguishing between samples at higher taxonomic levels [28].

Admittedly, there are other alternative species delineation methods, such as ABGD, K/θ, GMYC, PTP, and Haplowebs (introduced by Fontaneto et al. [29]). The molecular threshold method was chosen here, as it is one of the simplest methods available for taxa identification; however, it is imperfect [6]. The *best threshold method* proposed by Lefébure [9] was designed to for use in species delimitation; by using this method, the only molecular threshold (“best threshold”) for differentiation between two taxonomic ranks is defined. Similarity-based or BLAST-based methods have commonly been used for classification when nuclear ribosomal RNA genes and their internal transcribed spacer regions are selected as markers [30–32]. Studying small sections of 18S rRNA genes would benefit studies limited by sequence length, such as next generation sequencing, as these are limited to segments <400bp. In the present study, we aimed to test the correlation of the 18S rRNA gene as a marker for the taxonomic identification of copepods using (1) different regions of the gene, and (2) using the “best threshold.” Distributions of 18S rRNA gene sequence similarities at four taxonomic levels (species, genus, family, and order within the subclass Copepoda) were investigated for the test. To improve taxonomic accuracy, we used probabilistic methods to estimate the accuracy of taxonomic identifications and proposed the lowest similarity threshold to be used.

## Materials and Methods

### 2.1 Sequence retrieval and alignment

All sequences used in this test were published and made available in public sequence databases. A total of 895 18S rRNA gene sequences of copepods (with the exclusion of environmental samples) were acquired from GenBank on March 28, 2014 (S1 Table). To facilitate further analysis, all sequences were labeled with special ID numbers (representing the sample’s identification by Order, Family, Genus, and Species, according to the taxonomic database of NCBI). To ensure data quality, sequences not associated with published literature or found to contain ambiguous sites were removed. After screening, a total 531 published sequences (including 384 species, 203 genera, 84 families, and 7 orders) of the right taxonomy and covering the target regions were analyzed (S2 Table). It is known that the reliability of the sequences from GenBank is questionable [33], and misidentification of the sequenced specimen does occur (e.g., HQ008753, cf. [34]). Therefore, literature associated with these 531 sequences was carefully checked, and a subset of 189 sequences (S3 Table), published in taxonomic and phylogenetic studies which included species descriptions, were further analyzed. Sequence alignment was performed on the web server GUIDANCE [35] with its default parameters, using MAFFT version 5 [36] as the alignment algorithm.

### 2.2 Divergences of variable and conserved regions

The identification of variable regions was based on the sequence alignments of 192 Copepoda species (including 8 *Acartia* copepods and 184 other copepods), and various eukaryotic outgroup species, including the ciliate *Tetrahymena setosa* (Eukaryota, Alveolata, Ciliophora, Intramacronucleata, Oligohymenophorea, Hymenostomatida, Tetrahymenina, Tetrahymenidae; GenBank Accession No. AF364041), the insect *Drosophila melanogaster* (Eukaryota, Metazoa, Ecdysozoa, Arthropoda, Hexapoda, Insecta, Pterygota, Neoptera, Endopterygota, Diptera, Brachycera; GenBank Accession No. KC177303), and the crustacean *Bosmina longirostris* (Eukaryota, Metazoa, Ecdysozoa, Arthropoda, Crustacea, Branchiopoda, Diplostraca, Cladocera, Anomopoda, Bosminidae; GenBank Accession No. Z22731). To help with determination of variable regions, the calanoid copepod 18S rRNA secondary structure model described by Wang [37] was also analyzed. The genus *Acartia* was found to be highly divergent from other copepods, and greatly influenced the sequence alignments of Copepoda as a whole, especially with respect to the variable regions. The 8 species in the genus *Acartia* excluded from subsequent analyses of variation and genetic distance were nevertheless taken into account during the identification of variable regions. Excluding *Acartia* species, an entropy plot was calculated using the sequence alignments of the other 184 Copepoda species (S4 Table), via BioEdit 7.0.0 [38]. Because most of the 184 sequences were incomplete at either end, after trimming, there were 97 and 65 sequences with relatively complete 5′ and 3′ ends. The variability of the 5′- and 3′-ends was calculated based on the alignments of these 97 and 65 sequences, respectively. The molecular divergences of both the variable and conserved regions were calculated using MEGA version 5.0 [39]. The pairwise genetic distance was calculated using *p*-distance, which Collins et al. [40] suggested should be used for specimen identification.

### 2.3 Determination of target sections

All 18S rRNA gene sequences were aligned, and nearly-whole-length sequences that include all variable regions (V1-9) were extracted for statistical analysis. Five 18S sequence sections used in biodiversity assessments were chosen for analysis as well. These sections were of different lengths and contained different variable regions (V1-3, V4-5, V7, V8, and V9). Primers matching these sites were used to identify the sections from the alignment file. Sequences containing these sections were extracted and stored as separate FASTA databases. As most of the 18S rRNA gene sequences were incomplete at both ends, the lack of a few end sites was allowed in Sections 1 (V1-3), and 5 (V9), but only if the variable regions were complete. Sequences lacking confirmed species names should be used with caution, because they could be confused with other sequences in the same taxonomic category. For instance, if there is a sequence with a certain genus (or family) name, but with the identification to species (or genus) is uncertain, in this case, we could not judge whether this sequence is the same species (or genus) as other sequence in this genus (or family). Thus, sequences with uncertain identifications were not be used, in case it is not the only sequence in its taxonomic category. However, we wish to retain useful information whenever possible. Thus, we retained only one sequence of uncertain species origin in each genus without any confirmed sequences; sequences of uncertain origin at the genus level or higher were excluded from the statistical analysis, as there were other confidently named sequences.

### 2.4 Sequence similarities of target sections

The pair-wise sequence similarities of each sequence section were calculated using the Basic Local Alignment Search Tool (BLAST, version: ncbi-blast-2.2.28+) [41]. First, the FASTA databases of the sequence sections were formatted using the ‘makeblastdb’ program [42], then used to conduct a BLAST search. The FASTA databases were then used as query sequence files during the ‘blastn’ searching of the formatted databases, according to the output parameter output: outfmt = 6 (give a tabular output of query id, subject id, % identity, alignment length, mismatches, gap opens, q. start, q. end, s. start, s. end, evalue, and bit score). ‘% identity’ is the similarity value between the query and subject sequences. Here the ‘query id’, ‘subject id’, and ‘% identity’ were used in the statistical analysis. Prior to analysis, invalid data from mismatched and short matched sequences should be removed. As a typical example of a mismatch, the result of a comparison between nearly-whole-length sequences A (Accession No. AY626994.1) and B (Accession No. EU380295.1) was partly as follows: % identity = 89.47, alignment length = 19, q.start = 641, q.end = 659, s.start = 1728, s.end = 1710. Here sites 641–659 in sequence A were mismatched with the sites 1728–1710 in sequence B. In another case, a short matched result resulted from the comparison of sequence C (Accession No. AY627029) and D (Accession No. JF781547), with a partial output: % identity = 92.31, alignment length = 26, q.start = 1742, q.end = 1767, s.start = 1743, s.end = 1768. Usually alignment length would be a useful means of distinguishing mismatch and short match data from valid data. For sections 1, 2, and 4, blast results were sorted by alignment lengths, and values originating from mismatched and short matched sequences were identified by their shorter alignment lengths and removed from the analysis, and 100% of the expected data (number of sequences pairs) was retained. However, this did not work with the results of section 3 and 5, where some short matched results could not be distinguished from normal matched results based on alignment lengths, presumably because of the high variability and short lengths of these sections themselves. As manually checking thousands of sequences or more was unfeasible, the cut-off alignment lengths were designated at 100 (67 was the highest value below 100) and 104 (63 was the highest value below 104), for sections 3 and 5, respectively. Similarity values with alignment lengths shorter than the cut-off values were removed. As the result, 99% and 97% of the expected data respectively for section 3 and 5 were retained.

### 2.5 Statistics of sequence similarities

The sequence similarities were sorted into five categories: intra-specific similarities (S), inter-specific but intra-generic similarities (G), inter-generic but intra-familial similarities (F), inter-familial but intra-order similarities (O), and inter-order similarities (I). The distribution of these categories of similarities was plotted by sections, using boxplots with SPSS 16.0 (SPSS Inc., Chicago, IL). The cumulative frequency distribution of similarities was calculated and plotted using Microsoft Office Excel 2007. The best threshold for delimiting each different distribution of the five categories was carried out according to the method described by Lefébure et al. [9]. Briefly, the cumulative frequency distribution curves of the similarities that were above (positive curve) or below (negative curve) a range of thresholds were plotted on the same graph. The best similarity threshold was the value where the positive curve of a category (e.g., S-P in S1 Fig) and the negative curve of the adjacent category (e.g., G-N in S1 Fig) crossed.

The following probability formulas were used to estimate the accuracy of the specific lower similarity threshold for the sorting of samples into different taxonomic categories:

$$SP_{\left(S\geq n\right)}=(1-ICP_{\left(S\geq n\right)})\times (1-OCP_{\left(S\geq n\right)})\times (1-FCP_{\left(S\geq n\right)})\times (1-GCP_{\left(S\geq n\right)})$$ (1)

$$GP_{\left(S\geq n\right)}=(1-ICP_{\left(S\geq n\right)})\times (1-OCP_{\left(S\geq n\right)})\times (1-FCP_{\left(S\geq n\right)})$$ (2)

$$FP_{\left(S\geq n\right)}=(1-ICP_{\left(S\geq n\right)})\times (1-OCP_{\left(S\geq n\right)})$$ (3)

$$OP_{\left(S\geq n\right)}=(1-ICP_{\left(S\geq n\right)})$$ (4)

where *S* is the similarity between the query and the subjected sequences, and *n* is the lower threshold of sequence similarity. *SP*_(S ≥ n)_, *GP*_(S ≥ n)_, *FP*_(S ≥ n)_, and *OP*_(S ≥ n)_ represent the probability that the query and subjected sequences, with similarities of no less than *n* (*S* ≥ *n*), belong to the same category of species, genus, family, and order, respectively. *ICP*_(S ≥ n)_, *OCP*_(S ≥ n)_, *FCP*_(S ≥ n)_, and *GCP*_(S ≥ n)_ are the cumulative frequencies of sequence similarities in the four categories (I, O, F, and G, respectively) above the similarity threshold of *n* (*S* ≥ *n*). This probability was considered to be the rate of accuracy for each similarity threshold (*n*) when identifying samples at each taxonomic level (intra-species, intra-genus, intra-family, intra-order, and inter-order).

## Results

### 3.1 V2, V4, V7, and V9 are candidate regions for use in the study of copepod phylogeny and biodiversity

**Fig 1:**
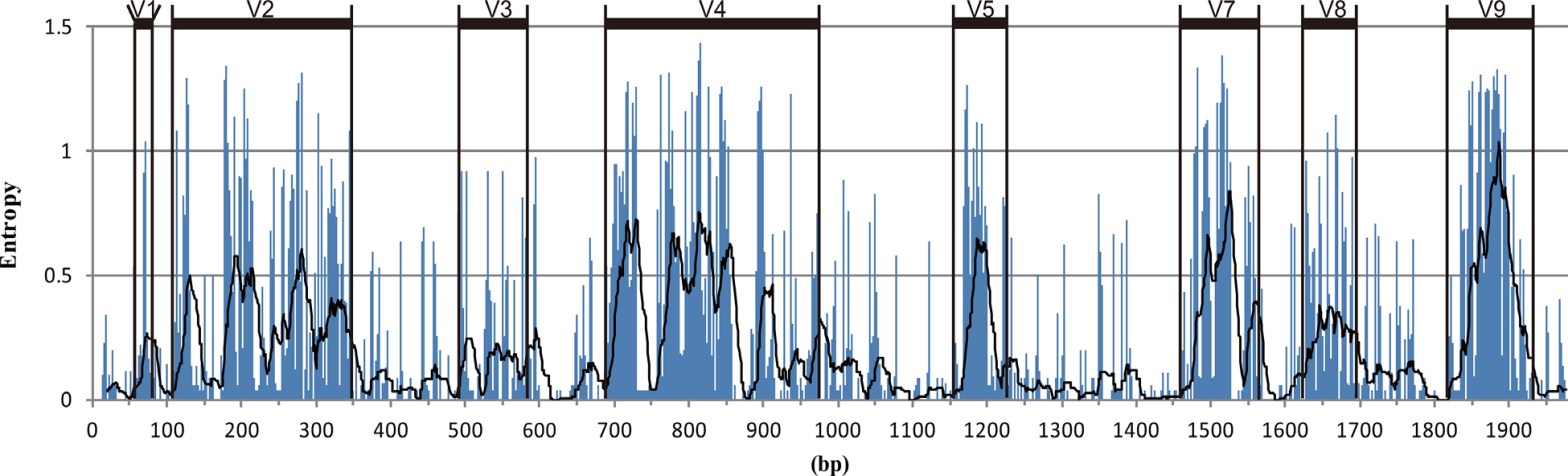
Entropy plot calculated based on site variability in an alignment of 184 copepod 18S rRNA genes, and the distribution of variable regions. A trend line represents the mean variability for successive windows of 20 positions.

Most of the nuclear 18S rRNA gene copepod sequences retrieved from GenBank are incomplete at the 5′- and/or 3′-ends. Nevertheless, both ends are considered conserved, based on the alignment of relatively complete sequences (Fig 1). After trimming off the two ends, the 184 sequences of the 18S rRNA gene range from 1694 to 1764 bp and include eight variable regions (V1–7, and V9) separated by linker regions (Table 1). The variable regions were marked on the secondary structure of Copepod 18S rRNA (S2 Fig). The greatest variations in length are in V7, especially in the genus *Acartia*. In this genus, V7 ranges from 86 to 141 bp in length, and is thus much longer than that of other copepods (74–93 bp). This length expansion of V7 leads to the formation of a special secondary structure in the loop region of Helix 43 [37]. V4 (213–256 bp) and V2 (184–214 bp) both vary significantly in length as well.

**Table 1:** Sequence characteristics of the 18S rRNA gene from 8 species in the genus *Acartia*, and 184 other copepod species.

Molecules	Length of *Acartia* (bp)	Length (bp)	GC	Nt	Nv	Nc	PI(%PI)
18S	1,689–1,787	1,694–1,742	48.5	1,878	974	854	784 (41.7)
TCs	849–869	859–862	49.1	864	258	603	178 (20.6)
CVs	829–938	834–881	47.8	1,014	716	251	606 (59.8)
V1	12–23	22–24	41.3	24	21	3	18 (75)
V2	184–214	189–213	45.9	245	178	59	149 (60.8)
V3	91–92	90–93	47.3	96	55	38	45 (46.9)
V4	213–242	219–256	45.9	286	188	70	165 (57.7)
V5	71–75	70–74	53.9	75	64	11	52 (69.3)
V7	86–141	74–93	48.0	100	68	30	57 (57)
V8	62–67	62–68	54.4	76	51	21	44 (57.9)
V9	85–97	91–98	49.4	112	91	19	76 (67.9)

18S, copepod 18S rRNA genes with the two ends trimmed (all the variable regions were intact); TCs, Total conserved region sequences in 18S; CVs, combination of all variable regions; V1-9, variable regions; GC, GC contents; Nt, total number of sites compared; Nc, total number of conserved sites; Nv, total number of variable sites; PI, parsimony-informative sites.

The percentage of parsimony-informative sites (%PI) partly reflects the variability of the V regions (Table 1). The top 75% of the PI sites were found in the shortest variable region, V1, followed by 69.3% in V5, 67.9% in V9, and 60.8% in V2. The entropy plot of the 18S rRNA genes alignment presents a visualization of this variability (Fig 1). The most diverse sites are concentrated in the V9 region. The largest portion of variable sites is divided between the V2 (Nv = 178) and V4 (Nv = 188, Table 1) regions.

**Fig 2:**
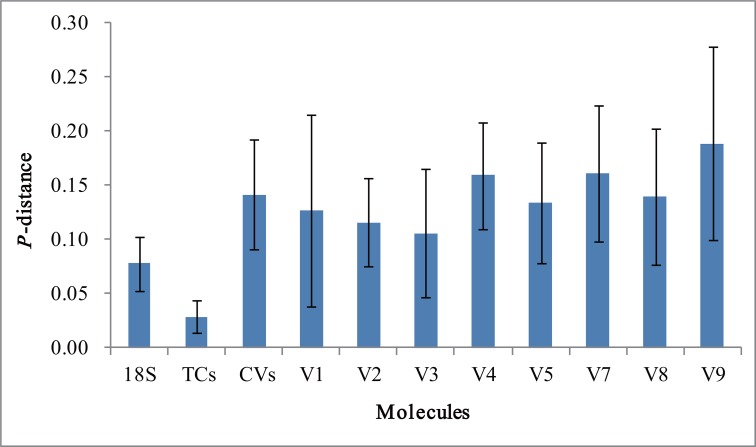
A comparison of the nucleotide divergences of the copepod 18S rRNA genes of different regions. 184 18S rRNA genes representing 184 copepod species (excluding species in the genus *Acartia*) were used to calculate pairwise genetic distances using *p*-distance. Values are shown as means ± SD. Variable names are consist with Table 1.

The nucleotide divergence of the V regions was further evaluated using pairwise genetic distance statistics (Fig 2). Variable regions are obviously more divergent than core regions and nearly-whole-length 18S rRNA gene. The average inter-specific distances are highest in V9 (0.189, SD = 0.090), V7 (0.162, SD = 0.063), and V4 (0.159, SD = 0.049). The divergences of these three V regions are higher those of all eight regions combined (0.18, SD = 0.063).

### 3.2 Choice of tested sections

To evaluate the taxonomic resolution of the 18S rRNA gene, nearly-whole-length sequences (ranging from 1701 to 1851 bp) and several shorter sections were tested individually. Information about these 18S rRNA gene sections and nearly-whole-length sequences, such as their V regions, lengths, primers, and taxa statistics are listed in Table 2. The primer binding sites are also noted in a depiction of the secondary structure of copepod 18S rRNA (S2 Fig). These sections vary in length from about 100–600 bp, and each contains 1–3 V regions. Primers used to amplify these sections were taken from papers describing studies of eukaryotic diversity. Sections 1, 2, 4, and 5, amplified with primers used in other biodiversity assessments [27,29,32,37], contain V1-3, V4-5, V8, and V9, respectively. As the length of V7 was found to be particularly divergent, and exhibited a high degree of nucleotide variability, section 3, which includes V7, was also tested, though the primers [33] designed for this section had not previously been used in biodiversity studies.

**Table 2:** Copepod 18S rRNA genes used in the statistical similarity analysis.

18S rRNA genes	V regions	Lengths of V regions (bp)	Lengths of Sections (bp)	Primers pairs for Sequence amplification	Number of Sequences	Number of orders	Number of families	Number of genus	Number of species
Nearly-whole-length	V1-9	738–785	1701–1851		226	7	70	134	185
Section 1	V1-3	302–327	481–544	Euk1A [43], Euk516 [43]	320	7	76	169	265
Section 2	V4-5	291–329	537–612	Ami6F1 [24], Ami9R [24]	410	7	82	179	309
Section 3	V7	83–108	125–150	#3 [44], #4_RC [44]	300	7	79	177	284
Section 4	V8	62–68	158–165	F–P [45], R–P [45]	390	7	82	178	301
Section 5	V9	91–98	107–139	1391F [27], EukB [27]	307	7	80	172	262

### 3.3 The distribution of sequence similarities

**Fig 3:**
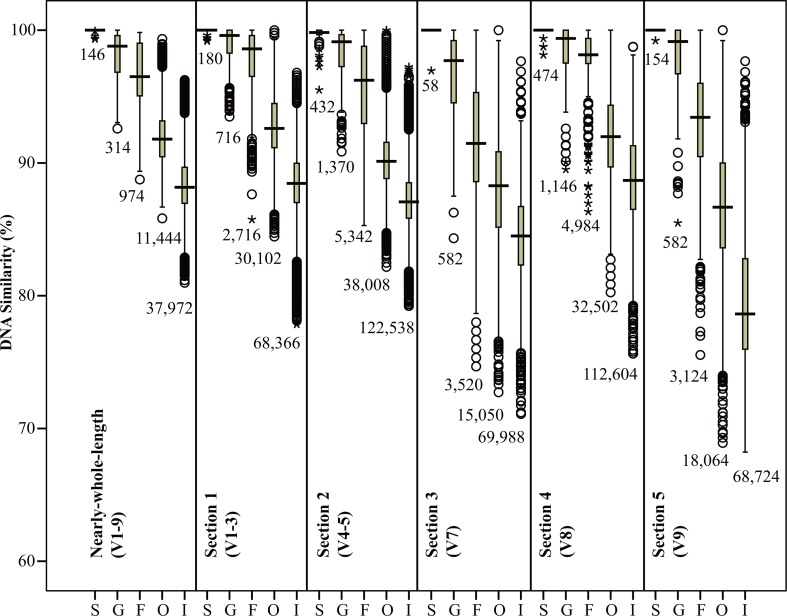
The boxplot distribution of sequence similarities for different 18S rRNA gene regions in the intra-species (S), inter-species but intra-genus (G), inter-genus but intra-family (F), inter-family but intra-order (O), and inter-order (I) categories. The central boxes represent the middle three quarters of the data. The total number of samples in each plot is listed at the bottom of each box.

Overall, the similarities between copepod 18S rRNA gene pairs (except for those in the genus *Acartia*) range from 68% to 100%, and gradually decrease with the broadening of taxonomic rank (S > G > F > O > I, Fig 3), with a small overlap remaining at the broadest level. The range of similarity is the narrowest for nearly-whole-length sequences (81% to 100%) compared among 5 sections. At the species level, intra-species (S) similarities are nearly 100% in all sections tested. Distribution boxes are always separated between F and O except that in section 3 (V7), while G could hardly be separated from F except in section 5 (V9). Distributions among other taxonomic categories vary with sections (Fig 3).

A total of 13 nearly-whole-length sequences (Accession No. GU969156, GU969157, GU969195-98, and JX995285-91), including those of 8 species of *Acartia*, were separately analyzed. The intra-specific similarity within two species (*Acartia bifilosa* and *Acartia tonsa*) was 100%, while the inter-specific similarities within the genus *Acartia* ranged from 74.77% to 86.56%, with an average inter-species similarity of 78.79%. This is significantly lower than that exhibited by other copepod genera (92.59–100% similarity, with an average of 98.17%). The similarity of the 8 species within the genus *Acartia* with copepod species of other genera in the subclass Copepoda (average similarity of 80.49%) is lower than that observed between other non-*Acartia* Copepoda species, which is even lower than the similarity between species of Copepoda belonging to different orders (range from 80.97% to 96.25%, 88.38% on average). Thus, it is not feasible to use sequence similarity methods to reveal the species relationships within the genus *Acartia* or for the elucidation of the higher taxonomic classification of *Acartia* species.

### 3.4 Best threshold values for taxonomic success

Lefébure et al. [9] proposed that the best threshold value of molecular divergence be used to discriminate between two different taxonomic ranks. The best threshold value is associated with the best compromised rate of successful identification, which depends on the distance between two taxonomic ranks. Success rates are divided into the following grades: rates between 50 and 60% indicate that the two ranks are difficult to distinguish, 60 and 80% success indicates that the ranks can be distinguished in a basic sense, better distinguishing occurred between success rates of 80 and 90%, while 95% or higher was considered a perfect success rate [9].

In this study, the best similarity thresholds and their corresponding success rates for taxonomic classification were revealed via analysis of the overlap of distributions of different ranks (Table 3). When distinguishing between taxonomic rank S (intra-species) and G (inter-species but intra-genus), the best similarity thresholds were found in nearly-whole-length sequences and sections 2, 3 and 5, but all close to 100% (Table 3) and close to the rate of potential PCR errors. A low overall success rate ranging from 62 to 73% in attempts to discriminate between G and F (inter-genus but intra-family) was observed in all sections except section 5 (Table 3). Nearly-whole-length sequences and other sections besides section 3 (V7) are useful for differentiating between F and O (inter-family but intra-order) with a relatively high rate of success (ranging from 78 to 89%). Most of the sections differentiate between O and I (inter-order) with nearly an 80% rate of success, while low rates of success (68%) were observed for sections 3 (V7) and 4 (V8).

**Table 3:** The best and lowest similarity thresholds for taxonomic resolution.

Sequences	V regions	The best similarity thresholds/success (%)				The lowest similarity thresholds/accuracy (%)*			
		S/G	G/F	F/O	O/I	Intra-species	Intra-genus	Intra-family	Intra-order
Nearly-whole-length	V1-9	99.9/93	98.4/64	94.2/86	90.2/81	100/97	100/100	97/96	93/96
Section 1	V1-3		99.4/63	95.4/82	90.6/82			98/95	93/96
Section 2	V4-5	99.7/66	97.8/67	92.6/83	88.6/78		100/99	97/96	92/95
Section 3	V7	99.3/82	94.6/73	89.9/68	86.0/68		100/96	96/97	91/96
Section 4	V8		99.8/62	95.7/89	90.6/68			98/96	95/97
Section 5	V9	99.3/67	96.6/79	90.2/78	83.2/77		100/95	96/96	90/96

* 95% is the lowest accuracy accepted in this case.

### 3.5 Lowest thresholds for taxonomic accuracy

The best threshold method suggested by Lefébure et al. [9] tries to achieve the best compromise between the accuracy and success rate of taxonomic classification. However, we are much more concerned about the rate of accuracy. We tried to use the lowest threshold to ensure an acceptable rate of accuracy.

According to the statistic of probability (S5 Table), there is a 97% probability that 100% of the similar sequences of nearly-whole-length 18S rRNA gene belong to the same species (intra-species), and a 100% probability that they belong to the same genus (intra-genus, whether intra- or inter-species). Similarly, sequences with similarities greater than 99% would belong to the same species with a probability of only 24%, and have a 66% probability of belonging to the same genus. In other words, 24% and 66% are the rates of accuracy for the classification of samples at the species and genus levels, at the lowest similarity threshold, 99%. If accuracies no lower than 95% are acceptable (Table 3), 100% similarity is the lowest threshold for the nearly-whole-length 18S rRNA gene in the classification of copepods at the species (97% accuracy) and genus level (100% accuracy), while similarities of 97% and 93% are, respectively, the lowest acceptable thresholds at the family and order levels. For all 18S rRNA gene sections tested in this study, no similarity threshold provides greater than 95% accuracy at the species level. Sections 2 (99% accuracy), 3 (96% accuracy), and 5 (95% accuracy) can be used to classify a sample at the genus level when there is 100% similarity. All sections can be used to classify a sequence to family and order, with the lowest similarity thresholds ranging from 90% to 98%.

### 3.6 Sequence reliability

Misidentification of the species source of the sequences acquired from GenBank could affect the distance between taxa and lower the success rate of identification. To verify the distances between taxonomic ranks, additional tests were performed based on the subset sequences from the associated taxonomic studies, which we assumed to be reliable (S3 and S6 Tables). A similar pattern was observed as in the results, with sequence similarity gradually decreasing with the broadening of taxonomic rank (S > G > F > O > I, S3 Fig), while the similarity thresholds are little changed (S7 Table); distances (S3 Fig) and identification success rates (S7 Table) increased between G and F, and reduced between O and I. However, there was essentially no change between F and O; accuracies under a given threshold improved at the genus level and decreased at the order level (S8 Table comparing with S5 Table), which corresponds to the changes in success rates for G/F and O/I (S7 Table).

## Discussion

### 4.1 Taxonomic resolution of the 18S rRNA gene and comparison to mitochondrial genes

The complete 18S rRNA gene was considered effective for the species-level identification of calanoid copepods (Crustacea, Copepoda) [46]. However, some other studies did not support using 18S rRNA gene for species delimitation [28,47,48]. In this study, we found that the best similarity threshold when using the 18S rRNA gene for S/G discrimination is close to 100%, which is unrealistic when attempting to achieve a high rate of successful identification, owing to potential PCR or sequencing errors. However, considering that the 18S rRNA gene is highly intraspecifically conservative, it could be effective in species-level analyses based on the diagnosis of single nucleotide polymorphisms [49–51], and serve as an auxiliary tool when other data (such as morphological characteristics and other gene markers) are available [48,52–54].

Because of their high variability, V2, V4, V7, and V9 would be good candidates for use in studies of copepod phylogeny and biodiversity, as suggested by Hadziavdic et al. [55]. It appears that within the subclass Copepoda, the nuclear 18S rRNA gene is well-suited to use in classification at the family and order levels (success rates are more than or close to 80%); and compared with other variable regions, V9 is more valuable for resolutions at the genus level (success rates close to 80%). Many phylogenetic studies confirmed that the 18S rRNA gene is valuable for resolving relationships between copepods at the genus and family levels [56–65]. By using a Bayesian analysis based on the 18S rRNA gene, Huys et al. revealed that the order of Monstrilloida shares a common ancestor with the caligiform families within the order of Siphonostomatoida [66]. This gene was also used in phylogenetic analyses at higher levels of classification categories, such as Crustacea and Arthropoda [67–69]. However, it failed to resolve relationships between closely related species [47,70]. *Acartia* is the predominant genus of the family Acartiidae (Copepoda: Calanoida) [71]. The 18S rRNA genes of *Acartia* species are very divergent, both from other copepods and within the genus. Unfortunately, we have not found research that explains this phenomenon. However, V7 is highly divergent in the length of the 18S rRNA gene in Copepoda, and especially in the genus *Acartia* (ranges from 86 to 141 bp). The V7 forms a special secondary structure (in the loop region of Helix 43) in the genus *Acartia* [37], indicating the possible utility of the secondary structure of 18S rRNA for the phylogenetic study of *Acartia* copepods.

The mitochondrial gene COI has been widely used as a DNA barcode for species identification. Compared with the 18S rRNA gene, COI has a high resolution at species level and is also useful for revealing intraspecific variation [28,48,72,73]. Some researchers were also optimistic about the use of COI for taxonomic resolution at higher levels of classification [74–76]. However, in some cases, COI provides relatively poor resolution at higher taxonomic levels [7–9]. Moreover, the taxonomic resolution potential of COI and other DNA markers varies among organismal groups [77–79], and differences in sampling depth and analysis methods also affect analyses of taxonomic relationships [7,80–83].

Another mitochondrial gene, 16S rRNA gene, is more effective for taxonomic resolution at the genus level [75,76], but its use is not recommended because of some impediments to sequence alignment [9]. When secondary structure model is used, sequence alignment is expected to be more exact. Therefore, some sites that match to loops in the secondary structure would be hard to align and thus are removed. The same problems emerge during alignment of 18S rRNA gene; however, they are not obvious when sequence similarity, rather than genetic distance, is used for taxonomic resolution. This is because, when using sequence similarity, it is not necessary to compare a group of sequences all at once. There are other problems when comparing sequences using BLAST [84,85]. An obvious problem is that when two highly divergent sequences are compared, only part of the full length of the sequences could be matched. However, partially-covered similarity may be higher between two divergent sequences than wholly-covered similarity between a more similar pair of genes, confirming that similarity across an entire domain may be biologically more significant than short, almost exact matches [85]. Thus, it is necessary to control for query coverage (alignment length) when utilizing statistics of similarity.

### 4.2 Limits and prospects

Considering length limitation that is required by some research techniques, short sections of 18S rRNA gene were primarily used in biodiversity studies. Highly variable regions are more divergent than complete 18S rRNA genes and have larger genetic distances within and between some taxonomic ranks. However, there are some cases of 100% similarity for the sections between species and within higher taxonomic ranks (inter-genus and inter-family). Thus, taxonomic accuracy by using 18S rRNA gene sections would be lower than that by using the complete gene. For the nearly complete gene, there are also two cases of 100% similarity between species but within the genus; therefore, the complete 18S rRNA gene would misidentify closely related Copepoda species.

It has been suggested that conservative taxonomy should be prioritized [86]. Setting a lowest similarity threshold for taxonomic resolution is a conservative method. Query sequences with a relatively low rate of similarity to matched sequences could be classified at higher taxonomic levels. For instance, for a query sequence consisting of nearly whole-length 18S rRNA gene 98% similar to that of a matched sequence belonging to a known copepod, there is only an 18% probability that the sequence belongs to the same species, and a 57% probability that it belongs to the same genus as the known copepod. However, the sample can be identified at the family level with a 99% degree of accuracy (S5 Table). The price of demanding strict classification accuracy is a reduction of successful identification at lower taxonomic levels. Success rates are naturally determined by the divergence of molecules among taxonomic levels, but could be improved by expanding database coverage.

In this study, accuracy rates for taxonomic resolution are estimated based on publicly available 18S rRNA gene copepod (excluding *Acartia*) sequences, and without consideration of other taxonomic characteristics. Missing species, from which no sequence could be used in this test, would affect the distances within and between taxonomic ranks. Where highly divergent species were not sampled, interspecific distances and distances within taxonomic ranks would be underestimated. To estimate distance between lower and higher taxonomic ranks, underestimation of distance within the lower taxonomic rank could lead to overestimation of distance between the two taxonomic ranks; in contrast, underestimation of distance within the higher taxonomic rank could lead to underestimation of distance between the two taxonomic ranks. If a missing species is much divergent intra-genus (G), but the intra-familial distance between the missing species and other species in a different genus (F) may not be significantly greater than the average level, then the missing in sampling would lead to underestimation of distance within G (the lower rank) but not within F (the higher rank), thus distance between G and F would be overestimated; if the missing species is much divergent inter-order (I), but intra-order distance between this missing species and other species in a different family (O) were not significantly greater than the average level, then the missing in sampling would lead to underestimation of the distance within I (the higher rank) but not within O (the lower rank), and thus the distance between O and I would be underestimated. This could partially explain the changes in success rates when a subset of sequences instead of the entire dataset were analyzed, although the elimination of potentially misidentified sequences from this subsample also contributed to the increased success rates for G/F. In contrast, when closely related species were missing, interspecific distance was overestimated, and identification accuracy was reduced. Accuracy would be improved by (1) a growing body of 18S rRNA gene data for target groups, (2) accurate identification of the morphospecies sequences, (3) the use of other molecular data in combination with 18S rRNA gene, (4) the combination of 18S rRNA gene data with other biological characteristics such as morphology, phylogeny, habitat, and life history, and (5) the improvement of sampling techniques and data analysis methodology.

The use of fixed thresholds for taxonomy is not sufficient and could lead to misidentification [6,87]. Zhang et al. proposed the use of fuzzy membership to reduce misidentification [3]. The lowest similarity threshold proposed in this study aims to achieve a similar goal. Beside similarity-based and threshold methods used in this study, there are many other alternative methods that could be used in molecular taxonomy [88–92]. These methods for species delimitation are mainly based on three types of data: matrices of genetic distance, phylogenetic trees, and haplotype networks [29].

### 4.3 Hidden diversity

Because of the use of DNA markers, a great deal of hidden biodiversity has been revealed by researchers [93–97]. However, biodiversity may still be either underestimated or overestimated [98–100] because of the limits of sampling technology and data analysis methods. In addition, margins of error cannot be ignored. The OTU (operational taxonomic units) used in the estimation of biodiversity are usually identified based on empirical thresholds of molecular diversity. Thus, the definition of thresholds will directly affect estimation accuracy. For example, by using the V1-2 regions of 18S rRNA gene as molecular markers, the hidden biodiversity of marine zooplankton was revealed. Using a similarity threshold of 97% to identify OTU revealed copepod diversity to be twice that revealed by morphological identification [25]. The 18S rRNA gene region (which includes V1-2) used by researchers is similar in length to section 1 (which includes V1-3). Statistical analysis of section 1 indicates that the intra-specific similarity of copepods is near 100% (Fig 3), with individuals exhibiting sequence similarities no lower than 97%, and having a 96% chance of belonging to different species (S5 Table). Thus, using 97% as the threshold would obviously lead to an underestimation of copepod diversity.

As this analysis is limited to copepods, it is difficult to draw conclusions about whether the 18S rRNA gene variation pattern observed in this study occurs in all organisms. However, we speculate that the divergences behind regional variations in 18S rRNA gene are present in a variety of taxa. Machida and Knowlton [44] analyzed 11 metazoan taxa using a sliding window, and observed the highest nucleotide diversity to occur around the V1-2 regions. V2 is also the most divergent region in the 18S rRNA gene of dinoflagellates [101]. In contrast, V9 is the most divergent variable region in the copepod 18S rRNA gene. It is therefore unreasonable to use a single threshold to identify the OTU of all eukaryotic taxa. In order to improve the accuracy of estimation results, tailored thresholds for different taxa need to be determined via future analysis. As this relies on the expansion of database coverage, related analyses are expected to be more comprehensive and accurate.

## Conclusion

This study investigated the diversity of the 18S rRNA gene within the copepod subclass. Analysis of the variation between different 18S rRNA gene regions indicated that V2, V4, V7, and V9 are promising candidates for use in studies of copepod phylogeny and biodiversity. The best similarity thresholds and success rates calculated in this study revealed the potential of 18S rRNA gene for the facilitation of the resolution of a variety of taxonomic ranks within the subclass Copepoda. The lowest similarity threshold is suggested by this paper, in order to ensure the accuracy of sample identification.

## Supporting Information

S1 Fig Best similarity thresholds for the discrimination of each of the two categories (S/G, G/F, F/O, O/I) acquired by the analysis of the overlap in similarities among these categories. Taking the results of Nearly-whole-length as example. Positive growth curves of S-P, G-P, F-P, O-P, and I-P respectively represent the similarities between the frequency distributions of S, G, F, O, and I that accumulate with the reduction of similarity. The negative growth curves of G-N, F-N, O-N, and I-N represent the similarities between the frequency distributions of G, F, O, and I that accumulate with the increase of similarity. Values noted at the crossover point between the positive and inverse curves of each of the two categories represent the best similarity threshold and the corresponding success rate for the discrimination between the two categories.
(TIF)

S2 Fig Putative secondary structure of the 18S rRNA of calanoid copepods (modified from [37]). The locations of variable regions are indicated in gray. Maple leaves mark the start sites of the primers used for the amplification of the sequence sections (see Table 2).
(TIF)

S3 Fig Same as Fig 3, applied to the subset sequences. (TIF)

S1 Table List of 895 Copepod 18S rRNA sequence searched in GenBank. (XLSX)

S2 Table List of 531 Copepod 18S rRNA sequence used in similarity statistics. (XLSX)

S3 Table List of 189 subset 18S rRNA sequences from taxonomy related studies. (XLSX)

S4 Table List of 184 Copepod 18S rRNA sequences used in entropy plot. (XLSX)

S5 Table Accuracy of taxonomic identification at different similarity thresholds. (PDF)

S6 Table A subset of sequences from taxonomic studies, used in the additional analysis. (PDF)

S7 Table Same as Table 3, applied to the subset sequences. (PDF)

S8 Table Same as S7 Table, applied to the subset sequences. (PDF)
