# Supplementary figures and images for: Taxonomic Resolutions Based on 18S rRNA Genes: A Case Study of Subclass Copepoda
Source: PLoS One. 2015 Jun 24;10(6):e0131498. doi: 10.1371/journal.pone.0131498 (PMC4479608; doi:10.1371/journal.pone.0131498)

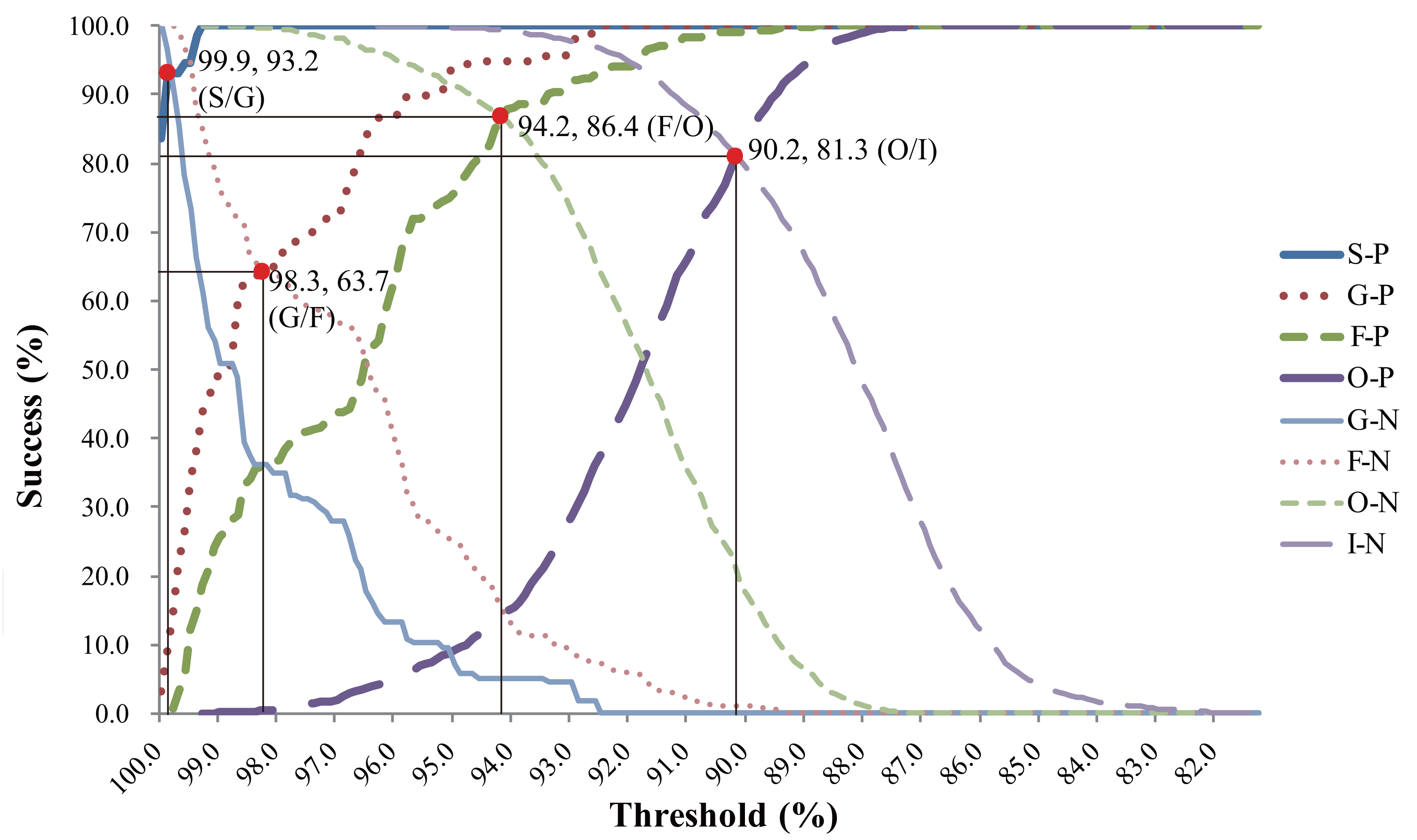

Supplement: S1 Fig — Taking the results of Nearly-whole-length as example. Positive growth curves of S-P, G-P, F-P, O-P, and I-P respectively represent the similarities between the frequency distributions of S, G, F, O, and I that accumulate with the reduction of similarity. The negative growth curves of G-N, F-N, O-N, and I-N represent the similarities between the frequency distributions of G, F, O, and I that accumulate with the increase of similarity. Values noted at the crossover point between the positive and inverse curves of each of the two categories represent the best similarity threshold and the corresponding success rate for the discrimination between the two categories. (TIF) [file pone.0131498.s001.tif]

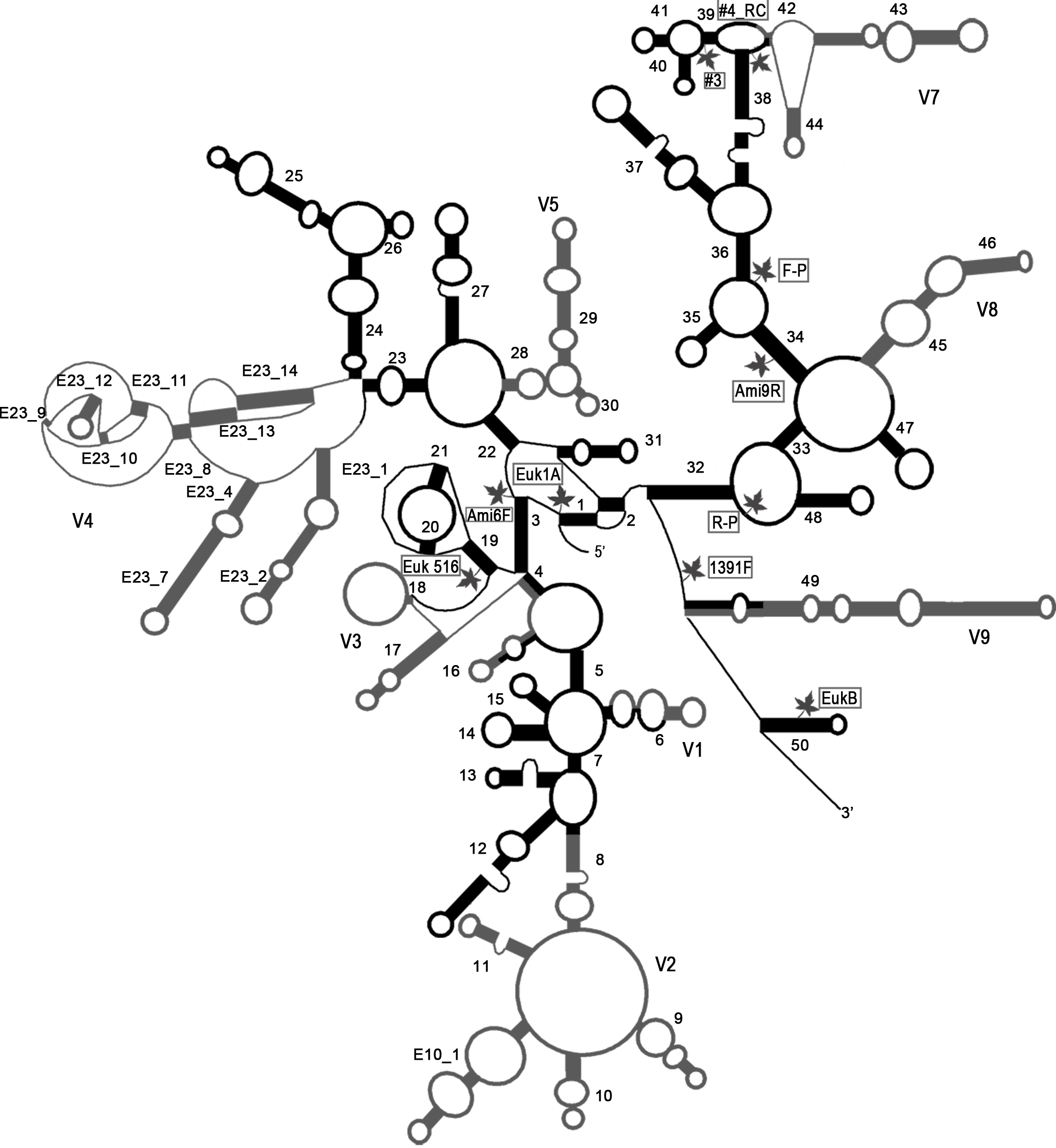

Supplement: S2 Fig — The locations of variable regions are indicated in gray. Maple leaves mark the start sites of the primers used for the amplification of the sequence sections (see Table 2). (TIF) [file pone.0131498.s002.tif]

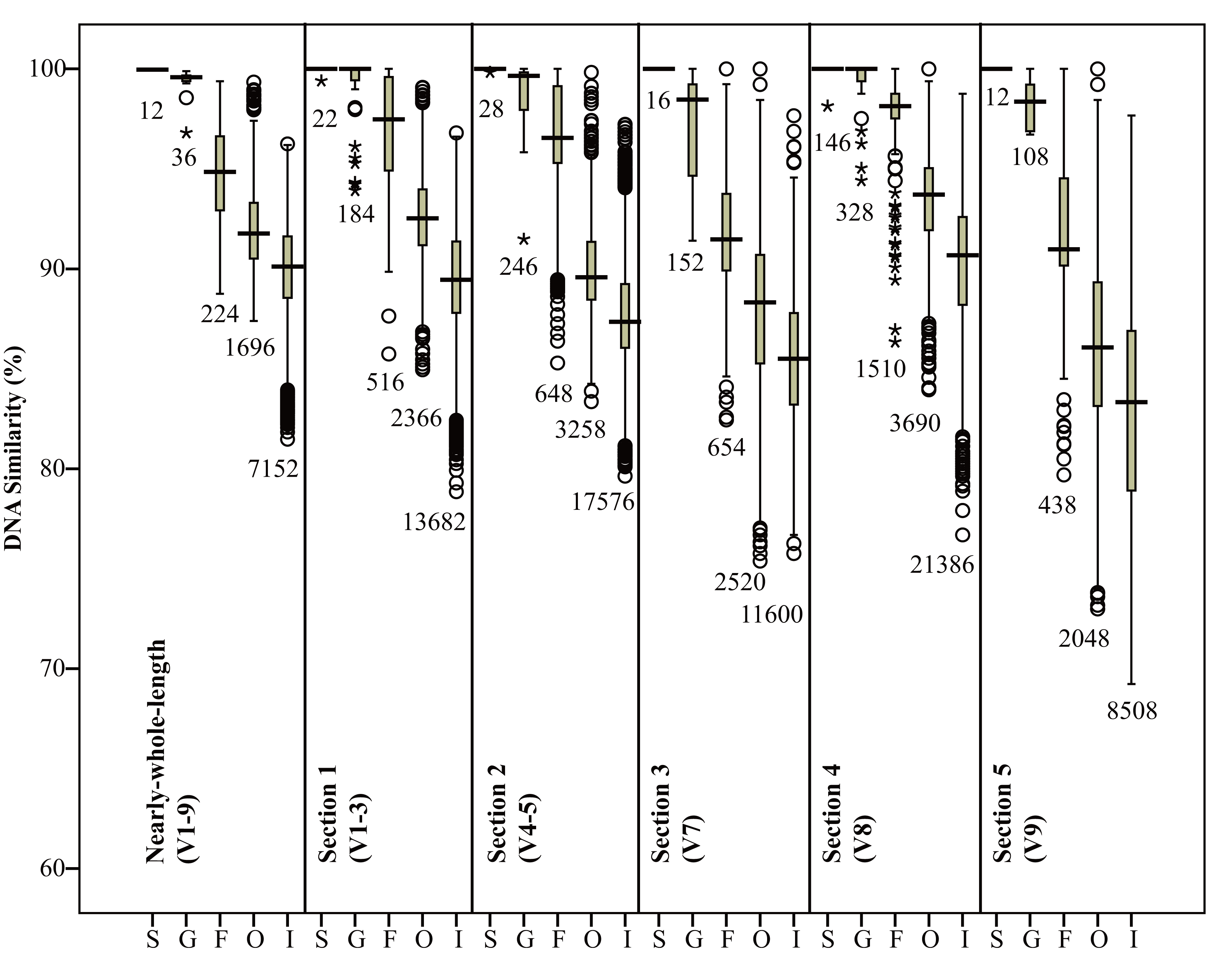

Supplement: S3 Fig — (TIF) [file pone.0131498.s003.tif]
