# Supplementary material for: Taxonomic Resolutions Based on 18S rRNA Genes: A Case Study of Subclass Copepoda
Source: PLoS One. 2015 Jun 24;10(6):e0131498. doi: 10.1371/journal.pone.0131498 (PMC4479608; doi:10.1371/journal.pone.0131498)
Supplement: S5 Table — (PDF) [file pone.0131498.s008.pdf]

S5 Table. Accuracy of taxonomic identification at different similarity thresholds.

Sequences	Categories	Lowest Similarity thresholds (%)																				
		100	99	98	97	96	95	94	93	92	91	90	89	88	87	86	85	84	83	82	81	80
Nearly-Whole-length	Intra-species	**0.968**	0.239	0.175	0.083	0.026	0.009	0.005	0.000	0.000	0.000	0.000	0.000	0.000	0.000	0.000	0.000	0.000	0.000	0.000	0.000	
	Intra-genus	**1.000**	0.658	0.571	0.481	0.259	0.173	0.091	0.042	0.012	0.002	0.000	0.000	0.000	0.000	0.000	0.000	0.000	0.000	0.000	0.000	
	Intra-family	**1.000**	**0.999**	**0.986**	**0.964**	0.927	0.877	0.780	0.607	0.404	0.211	0.073	0.015	0.001	0.000	0.000	0.000	0.000	0.000	0.000	0.000	
	Intra-order	**1.000**	**1.000**	**1.000**	**1.000**	**0.999**	**0.995**	**0.985**	**0.964**	0.914	0.835	0.726	0.561	0.361	0.171	0.063	0.024	0.011	0.004	0.000	0.000	
Section 1	Intra-species	0.634	0.133	0.076	0.035	0.011	0.003	0.000	0.000	0.000	0.000	0.000	0.000	0.000	0.000	0.000	0.000	0.000	0.000	0.000	0.000	0.000
	Intra-genus	0.918	0.487	0.358	0.226	0.154	0.081	0.037	0.021	0.009	0.002	0.000	0.000	0.000	0.000	0.000	0.000	0.000	0.000	0.000	0.000	0.000
	Intra-family	**0.999**	**0.989**	**0.948**	0.917	0.834	0.746	0.646	0.466	0.271	0.139	0.061	0.022	0.005	0.001	0.000	0.000	0.000	0.000	0.000	0.000	0.000
	Intra-order	**1.000**	**1.000**	**1.000**	**1.000**	**0.999**	**0.993**	**0.981**	**0.959**	0.909	0.818	0.688	0.509	0.326	0.169	0.087	0.045	0.024	0.012	0.005	0.002	0.001
Section 2	Intra-species	0.861	0.334	0.171	0.080	0.033	0.014	0.007	0.002	0.000	0.000	0.000	0.000	0.000	0.000	0.000	0.000	0.000	0.000	0.000	0.000	0.000
	Intra-genus	**0.989**	0.736	0.632	0.511	0.398	0.288	0.242	0.109	0.043	0.017	0.007	0.000	0.000	0.000	0.000	0.000	0.000	0.000	0.000	0.000	0.000
	Intra-family	**1.000**	**0.997**	**0.988**	**0.964**	0.940	0.908	0.859	0.807	0.705	0.515	0.333	0.154	0.058	0.014	0.002	0.000	0.000	0.000	0.000	0.000	0.000
	Intra-order	**1.000**	**1.000**	**1.000**	**1.000**	**0.999**	**0.998**	**0.994**	**0.981**	**0.954**	0.913	0.850	0.750	0.586	0.376	0.193	0.074	0.026	0.009	0.002	0.001	0.000
Section 3	Intra-species	0.784	0.615	0.404	0.326	0.267	0.149	0.116	0.080	0.033	0.012	0.009	0.003	0.000	0.000	0.000	0.000	0.000	0.000	0.000	0.000	0.000
	Intra-genus	**0.955**	0.927	0.858	0.817	0.761	0.649	0.592	0.521	0.399	0.234	0.173	0.120	0.060	0.037	0.019	0.008	0.003	0.001	0.000	0.000	0.000
	Intra-family	**1.000**	**0.998**	**0.991**	**0.985**	**0.972**	0.939	0.910	0.863	0.788	0.677	0.592	0.490	0.336	0.258	0.176	0.107	0.064	0.035	0.016	0.007	0.002
	Intra-order	**1.000**	**1.000**	**1.000**	**1.000**	**0.999**	**0.998**	**0.995**	**0.992**	**0.981**	**0.961**	0.938	0.897	0.813	0.744	0.630	0.495	0.386	0.272	0.182	0.122	0.065
Section 4	Intra-species	0.526	0.182	0.056	0.028	0.010	0.005	0.001	0.000	0.000	0.000	0.000	0.000	0.000	0.000	0.000	0.000	0.000	0.000	0.000	0.000	0.000
	Intra-genus	0.874	0.498	0.235	0.144	0.088	0.065	0.027	0.011	0.006	0.002	0.000	0.000	0.000	0.000	0.000	0.000	0.000	0.000	0.000	0.000	0.000
	Intra-family	**0.998**	**0.992**	**0.959**	0.912	0.824	0.765	0.609	0.437	0.331	0.207	0.115	0.063	0.029	0.013	0.004	0.001	0.000	0.000	0.000	0.000	0.000
	Intra-order	**1.000**	**1.000**	**0.999**	**0.994**	**0.978**	**0.966**	0.912	0.842	0.771	0.656	0.588	0.473	0.356	0.249	0.143	0.096	0.051	0.024	0.016	0.006	0.002
Section 5	Intra-species	0.642	0.430	0.250	0.151	0.109	0.075	0.059	0.033	0.016	0.010	0.006	0.003	0.000	0.000	0.000	0.000	0.000	0.000	0.000	0.000	0.000
	Intra-genus	**0.953**	0.914	0.836	0.760	0.673	0.591	0.502	0.379	0.293	0.193	0.128	0.077	0.049	0.028	0.020	0.012	0.009	0.004	0.002	0.001	0.000
	Intra-family	**1.000**	**0.999**	**0.990**	**0.981**	**0.962**	0.944	0.924	0.880	0.831	0.761	0.692	0.611	0.506	0.413	0.340	0.256	0.193	0.136	0.092	0.057	0.032
	Intra-order	**1.000**	**1.000**	**1.000**	**1.000**	**0.999**	**0.999**	**0.997**	**0.993**	**0.987**	**0.977**	**0.962**	0.936	0.911	0.883	0.854	0.811	0.776	0.743	0.702	0.650	0.572
