# Supplementary material for: Taxonomic Resolutions Based on 18S rRNA Genes: A Case Study of Subclass Copepoda
Source: PLoS One. 2015 Jun 24;10(6):e0131498. doi: 10.1371/journal.pone.0131498 (PMC4479608; doi:10.1371/journal.pone.0131498)
Supplement: S6 Table — (PDF) [file pone.0131498.s009.pdf]

S6 Table. A subset sequences from taxonomic studies used in additional analysis

18S rDNA sequences	Lengths (bp)	Number of Sequences	Number of order	Number of family	Number of genus	Number of species
Nearly-whole-length	1755-1843	96	7	51	87	93
Section 1	489-544	130	7	52	97	122
Section 2	574-612	148	7	56	105	138
Section 3	126-137	123	7	51	96	118
Section 4	160-165	165	7	55	103	139
Section 5	107-132	108	7	51	91	105
