# Supplementary material for: Taxonomic Resolutions Based on 18S rRNA Genes: A Case Study of Subclass Copepoda
Source: PLoS One. 2015 Jun 24;10(6):e0131498. doi: 10.1371/journal.pone.0131498 (PMC4479608; doi:10.1371/journal.pone.0131498)
Supplement: S7 Table — (PDF) [file pone.0131498.s010.pdf]

**S7 Table.** Same as Table 3, applied to the subset sequences.

| Sequences | V regions | The best similarity thresholds/success (%) | | | | The lowest similarity thresholds/accuracy (%)[*] | | | |
|---|---|---|---|---|---|---|---|---|---|
| | | S/G | G/F | F/O | O/I | Intra-species | Intra-genus | Intra-family | Intra-order |
| Nearly-whole-length | V1-9 | 99.9/83 | 98.5/94.4 | 93.1/71 | 91.1/66 | 100/100 | 99/95 | 97/97 | 94/95 |
| Section 1 | V1-3 | | 99.5/72.1 | 94.2/81 | 91.3/74 | | 100/97 | 97/96 | 95/98 |
| Section 2 | V4-5 | 99.9/79 | 99.1/69.9 | 92.6/84 | 88.8/71 | | 100/100 | 96/95 | 93/95 |
| Section 3 | V7 | 99.3/86 | 94.6/76.3 | 90.1/71 | 86.8/66 | | 99/96 | 95/95 | 92/95 |
| Section 4 | V8 | | 98.9/77.5 | 96.9/87 | 92.5/69 | | | 99/97 | 96/95 |
| Section 5 | V9 | 99.3/83 | 96.7/88.6 | 90.0/81 | 84.8/61 | | 99/98 | 94/95 | 92/96 |

[*] 95% is the lowest accuracy accepted in this case
