# Supplementary material for: Taxonomic Resolutions Based on 18S rRNA Genes: A Case Study of Subclass Copepoda
Source: PLoS One. 2015 Jun 24;10(6):e0131498. doi: 10.1371/journal.pone.0131498 (PMC4479608; doi:10.1371/journal.pone.0131498)
Supplement: S8 Table — (PDF) [file pone.0131498.s011.pdf]

S8 Table. Taxonomic acuracy estimated from the subset of 18S rDNA sequences.

Sequences	Categories	Lowest Similarity thresholds (%)																				
		100	99	98	97	96	95	94	93	92	91	90	89	88	87	86	85	84	83	82	81	80
Nearly-Whole-length	Intra-species	**1.000**	0.053	0.044	0.000	0.000	0.000	0.000	0.000	0.000	0.000	0.000	0.000	0.000	0.000	0.000	0.000	0.000	0.000	0.000	0.000	
	Intra-genus	**1.000**	**0.946**	0.797	0.676	0.548	0.376	0.251	0.121	0.034	0.006	0.001	0.000	0.000	0.000	0.000	0.000	0.000	0.000	0.000	0.000	
	Intra-family	**1.000**	**0.991**	**0.981**	**0.971**	0.916	0.859	0.740	0.542	0.315	0.142	0.043	0.009	0.000	0.000	0.000	0.000	0.000	0.000	0.000	0.000	
	Intra-order	**1.000**	**1.000**	**1.000**	**1.000**	**0.996**	**0.982**	**0.952**	0.879	0.725	0.567	0.395	0.243	0.140	0.080	0.053	0.036	0.015	0.003	0.000	0.000	
Section 1	Intra-species	0.400	0.088	0.053	0.045	0.016	0.008	0.000	0.000	0.000	0.000	0.000	0.000	0.000	0.000	0.000	0.000	0.000	0.000	0.000	0.000	0.000
	Intra-genus	**0.969**	0.580	0.489	0.412	0.295	0.179	0.076	0.042	0.015	0.002	0.000	0.000	0.000	0.000	0.000	0.000	0.000	0.000	0.000	0.000	0.000
	Intra-family	**1.000**	**0.992**	**0.978**	**0.957**	0.916	0.810	0.633	0.436	0.244	0.100	0.037	0.013	0.003	0.001	0.000	0.000	0.000	0.000	0.000	0.000	0.000
	Intra-order	**1.000**	**1.000**	**1.000**	**1.000**	**0.996**	**0.978**	0.943	0.878	0.758	0.632	0.505	0.371	0.220	0.118	0.068	0.046	0.030	0.013	0.006	0.001	0.000
Section 2	Intra-species	0.859	0.190	0.100	0.023	0.002	0.001	0.001	0.001	0.001	0.000	0.000	0.000	0.000	0.000	0.000	0.000	0.000	0.000	0.000	0.000	0.000
	Intra-genus	**0.997**	0.651	0.615	0.472	0.265	0.162	0.151	0.125	0.084	0.055	0.029	0.003	0.001	0.000	0.000	0.000	0.000	0.000	0.000	0.000	0.000
	Intra-family	**1.000**	**0.994**	**0.991**	**0.981**	**0.953**	0.920	0.875	0.794	0.698	0.545	0.375	0.172	0.077	0.021	0.004	0.000	0.000	0.000	0.000	0.000	0.000
	Intra-order	**1.000**	**1.000**	**1.000**	**0.998**	**0.995**	**0.988**	**0.976**	**0.951**	0.909	0.854	0.776	0.671	0.522	0.329	0.166	0.068	0.037	0.016	0.003	0.001	0.000
Section 3	Intra-species	0.843	0.519	0.356	0.340	0.300	0.099	0.053	0.037	0.005	0.000	0.000	0.000	0.000	0.000	0.000	0.000	0.000	0.000	0.000	0.000	0.000
	Intra-genus	**0.985**	**0.962**	0.933	0.891	0.844	0.753	0.677	0.570	0.398	0.200	0.129	0.073	0.027	0.013	0.006	0.002	0.001	0.000	0.000	0.000	0.000
	Intra-family	**0.998**	**0.992**	**0.987**	**0.981**	**0.972**	**0.947**	0.915	0.879	0.770	0.674	0.578	0.441	0.291	0.205	0.133	0.067	0.037	0.019	0.008	0.004	0.001
	Intra-order	**1.000**	**1.000**	**1.000**	**0.999**	**0.998**	**0.995**	**0.988**	**0.977**	0.949	0.913	0.877	0.807	0.702	0.625	0.494	0.360	0.274	0.186	0.116	0.073	0.033
Section 4	Intra-species	0.383	0.119	0.032	0.010	0.003	0.001	0.000	0.000	0.000	0.000	0.000	0.000	0.000	0.000	0.000	0.000	0.000	0.000	0.000	0.000	0.000
	Intra-genus	0.873	0.608	0.212	0.118	0.068	0.047	0.017	0.007	0.004	0.001	0.000	0.000	0.000	0.000	0.000	0.000	0.000	0.000	0.000	0.000	0.000
	Intra-family	**0.995**	**0.969**	0.913	0.855	0.766	0.640	0.407	0.234	0.135	0.054	0.029	0.011	0.004	0.002	0.001	0.000	0.000	0.000	0.000	0.000	0.000
	Intra-order	**1.000**	**0.999**	**0.997**	**0.985**	**0.954**	0.918	0.824	0.705	0.593	0.438	0.358	0.265	0.212	0.138	0.081	0.059	0.024	0.017	0.011	0.004	0.001
Section 5	Intra-species	0.830	0.547	0.237	0.000	0.000	0.000	0.000	0.000	0.000	0.000	0.000	0.000	0.000	0.000	0.000	0.000	0.000	0.000	0.000	0.000	0.000
	Intra-genus	**0.995**	**0.984**	0.916	0.869	0.809	0.726	0.695	0.599	0.461	0.240	0.125	0.070	0.039	0.019	0.015	0.009	0.007	0.003	0.001	0.000	0.000
	Intra-family	**1.000**	**0.998**	**0.993**	**0.992**	**0.984**	**0.975**	**0.951**	0.917	0.871	0.784	0.687	0.586	0.469	0.384	0.300	0.206	0.144	0.095	0.065	0.045	0.027
	Intra-order	**1.000**	**1.000**	**1.000**	**1.000**	**0.996**	**0.992**	**0.987**	**0.976**	**0.961**	0.933	0.890	0.838	0.779	0.720	0.666	0.582	0.515	0.454	0.388	0.336	0.277
